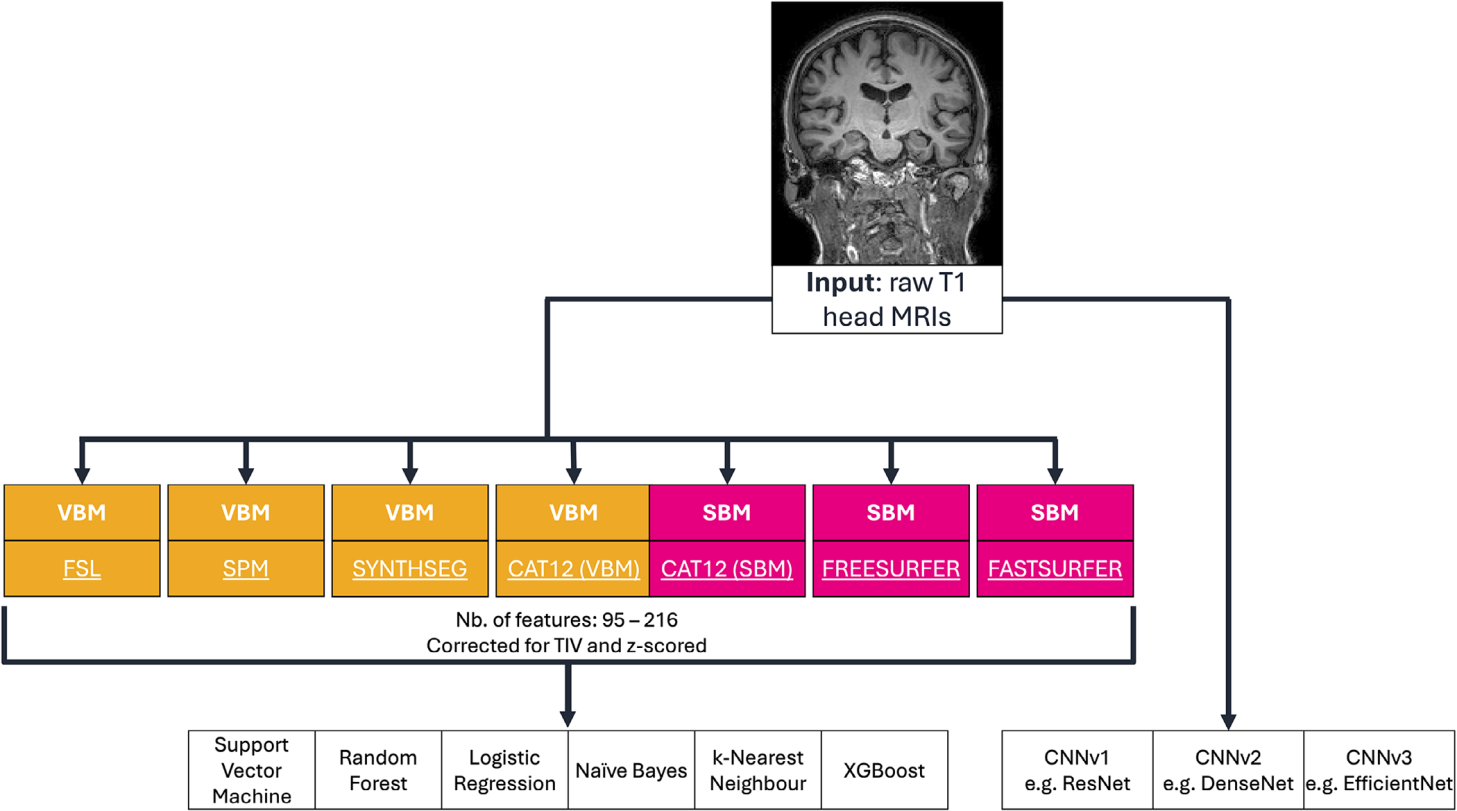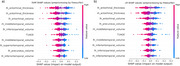# Systematic Comparison of MRI Preprocessing Pipelines and Classical Machine Learning Models for Dementia Classification: Benchmarking Against CNN Performance

**DOI:** 10.1002/alz70862_110254

**Published:** 2025-12-23

**Authors:** Anastasia Francoise L Gailly de Taurines, Gregory PT Scott, Paresh Malhotra, Payam Barnaghi

**Affiliations:** ^1^ UK Dementia Research Institute Centre for Care Research and Technology, London UK; ^2^ Imperial College London, Department of Brain Sciences, London UK; ^3^ Imperial College Healthcare NHS Trust, London UK; ^4^ UK Dementia Research Institute Centre for Care Research and Technology, London, London UK; ^5^ Imperial College London, Department of Brain Sciences, London, London UK; ^6^ Imperial College London, Department of Brain Sciences, London, Greater London UK; ^7^ UK Dementia Research Institute, Care Research and Technology Centre, London UK

## Abstract

**Background:**

Convolutional neural networks (CNNs) excel in classifying dementia subtypes from T1‐weighted MRI scans, yet their limited interpretability hinders clinical adoption. Classical machine learning (ML) models, while typically less performant, offer greater explainability by utilising predefined anatomically‐informed features. However, a systematic comparison of the many popular T1 MRI preprocessing pipelines for feature extraction — particularly in the context of dementia — is lacking.

**Method:**

This study evaluates seven widely used MRI preprocessing pipelines (FSL, SPM, CAT12 VBM, CAT12 SBM, SynthSeg, Freesurfer, FastSurfer) across five open‐source dementia imaging datasets (ADNI, OASIS, AIBLE, NIFD, NACC). Regional volumetric and surface‐based features extracted by each pipeline were input into six classical ML classifiers: support vector machines (SVM), random forest, logistic regression, naïve Bayes, k‐nearest neighbors (kNN), and XGBoost. Performance metrics, including accuracy, precision, sensitivity, and AUC‐ROC, were used to evaluate the classification of dementia subtypes (including Alzheimer’s disease and frontotemporal dementia). Additionally, raw MRI scans are classified using several 3D CNN architectures, including a modified ResNet18, to benchmark feature‐based ML models against CNN performance. The analysis pipeline is summarised in Figure 1.

**Result:**

Preliminary analysis on the ADNI dataset identified Freesurfer as the best‐performing preprocessing pipeline, achieving a mean accuracy of 0.872 across ML classifiers. Random forest emerged as the top‐performing model overall, with a mean accuracy of 0.840 across pipelines. Shapley value analysis for Freesurfer features revealed consistent influential regions across ML models; for example, Figure 2 highlights similarities between SVM and logistic regression models. The CNN results are currently being generated and will be available for comparison at the conference.

**Conclusion:**

This study highlights the strengths and limitations of different preprocessing‐ML combinations for dementia classification, providing a framework for optimising performance while enhancing interpretability. By systematically comparing these pipelines to CNNs, we aim to identify clinically viable alternatives that balance accuracy, explainability, and computational efficiency.